# Identification of potential autoantigens in anti-CCP-positive and anti-CCP-negative rheumatoid arthritis using citrulline-specific protein arrays

**DOI:** 10.1038/s41598-021-96675-z

**Published:** 2021-08-27

**Authors:** Thomas B. G. Poulsen, Dres Damgaard, Malene M. Jørgensen, Ladislav Senolt, Jonathan M. Blackburn, Claus H. Nielsen, Allan Stensballe

**Affiliations:** 1 Department of Health Science and Technology, Aalborg University, Fredrik Bajers Vej 5, 9220 Aalborg, Denmark; 2 Sino-Danish College (SDC), University of Chinese Academy of Sciences, Beijing, China; 3 Institute for Inflammation Research, Center for Rheumatology and Spine Diseases, Copenhagen University Hospital Rigshospitalet, Copenhagen, Denmark; 4 Department of Clinical Immunology, Aalborg University Hospital, Aalborg, Denmark; 5 Department of Clinical Medicine, Aalborg University, Aalborg, Denmark; 6 Institute of Rheumatology and Department of Rheumatology, 1st Faculty of Medicine, Charles University, Prague, Czech Republic; 7 Department of Integrative Biomedical Sciences and Institute of Infectious Disease and Molecular Medicine, University of Cape Town, Cape Town, South Africa

**Keywords:** Biomarkers, Rheumatology

## Abstract

The presence or absence of autoantibodies against citrullinated proteins (ACPAs) distinguishes two main groups of rheumatoid arthritis (RA) patients with different etiologies, prognoses, disease severities, and, presumably, disease pathogenesis. The heterogeneous responses of RA patients to various biologics, even among ACPA-positive patients, emphasize the need for further stratification of the patients. We used high-density protein array technology for fingerprinting of ACPA reactivity. Identification of the proteome recognized by ACPAs may be a step to stratify RA patients according to immune reactivity. Pooled plasma samples from 10 anti-CCP-negative and 15 anti-CCP-positive RA patients were assessed for ACPA content using a modified protein microarray containing 1631 different natively folded proteins citrullinated in situ by protein arginine deiminases (PADs) 2 and PAD4. IgG antibodies from anti-CCP-positive RA plasma showed high-intensity binding to 87 proteins citrullinated by PAD2 and 99 proteins citrullinated by PAD4 without binding significantly to the corresponding native proteins. Curiously, the binding of IgG antibodies in anti-CCP-negative plasma was also enhanced by PAD2- and PAD4-mediated citrullination of 29 and 26 proteins, respectively. For only four proteins, significantly more ACPA binding occurred after citrullination with PAD2 compared to citrullination with PAD4, while the opposite was true for one protein. We demonstrate that PAD2 and PAD4 are equally efficient in generating citrullinated autoantigens recognized by ACPAs. Patterns of proteins recognized by ACPAs may serve as a future diagnostic tool for further subtyping of RA patients.

## Introduction

Rheumatoid arthritis (RA) is a systemic autoimmune disease characterized by chronic inflammation of the joints and synovial tissue inflammation, leading to pain, swelling, bone erosion, and disability^1^. Approximately two-thirds of RA patients produce anti-citrullinated protein antibodies (ACPAs)^2,3^. These autoantibodies may be present years before the onset of clinical symptoms, underlining their possible involvement in the pathogenesis of early RA and may serve as early biomarkers^4,5^. Anti-CCP-positive and anti-CCP-negative RA can be regarded as two disease entities with different predisposing factors, etiology, disease severity, prognosis, and presumably pathogenesis^6,7^.

The conversion of peptidyl-arginine into peptidyl-citrulline, commonly known as protein citrullination or arginine deimination, is a posttranslational modification of proteins catalyzed by protein arginine deiminases (PADs). In mammals, five isoforms of PAD differ in tissue distribution and localization within cells: PAD1–4 and PAD6^8^. In particular, PAD2 and PAD4 are relevant to RA due to their expression in macrophages and neutrophils present in the synovial membrane of RA patients^9^. Their efficiency, relative to each other, in generating citrullinated neoepitopes recognized by ACPAs is not clear^10–12^. Citrullination is central to multiple regulatory cellular functions, such as cell differentiation, apoptosis, gene regulation, and inflammation^13^. Evidence is accumulating for a central role of citrullination in the pathogenesis of several diseases in addition to RA, including multiple sclerosis, Alzheimer’s disease, and cancers^14–16^.

The clinical presentation of RA varies considerably from patient to patient. In addition, the patients respond differently to various drugs. There is therefore an urgent need for the identification of new diagnostic tools to aid in patient stratification for precision medicine^17,18^. Characterization of ACPA autoantibody reactivity may provide new insight in this respect^19–21^. We have previously investigated the reactivity of autoantibodies against native proteins in RA patients and healthy subjects, demonstrating insignificant levels of autoantibody reactivity in especially healthy subjects for which intensities were significantly lower than both anti-CCP-positive and anti-CCP-negative RA patient samples against unmodified proteins^20^. Utilizing the high-throughput capacity of protein microarrays, we performed an exploratory study quantifying the binding of autoantibodies in plasma pools from well-characterized RA patients to more than 1600 different human proteins in citrullinated and non-citrullinated forms.

## Results

**Figure 1:**
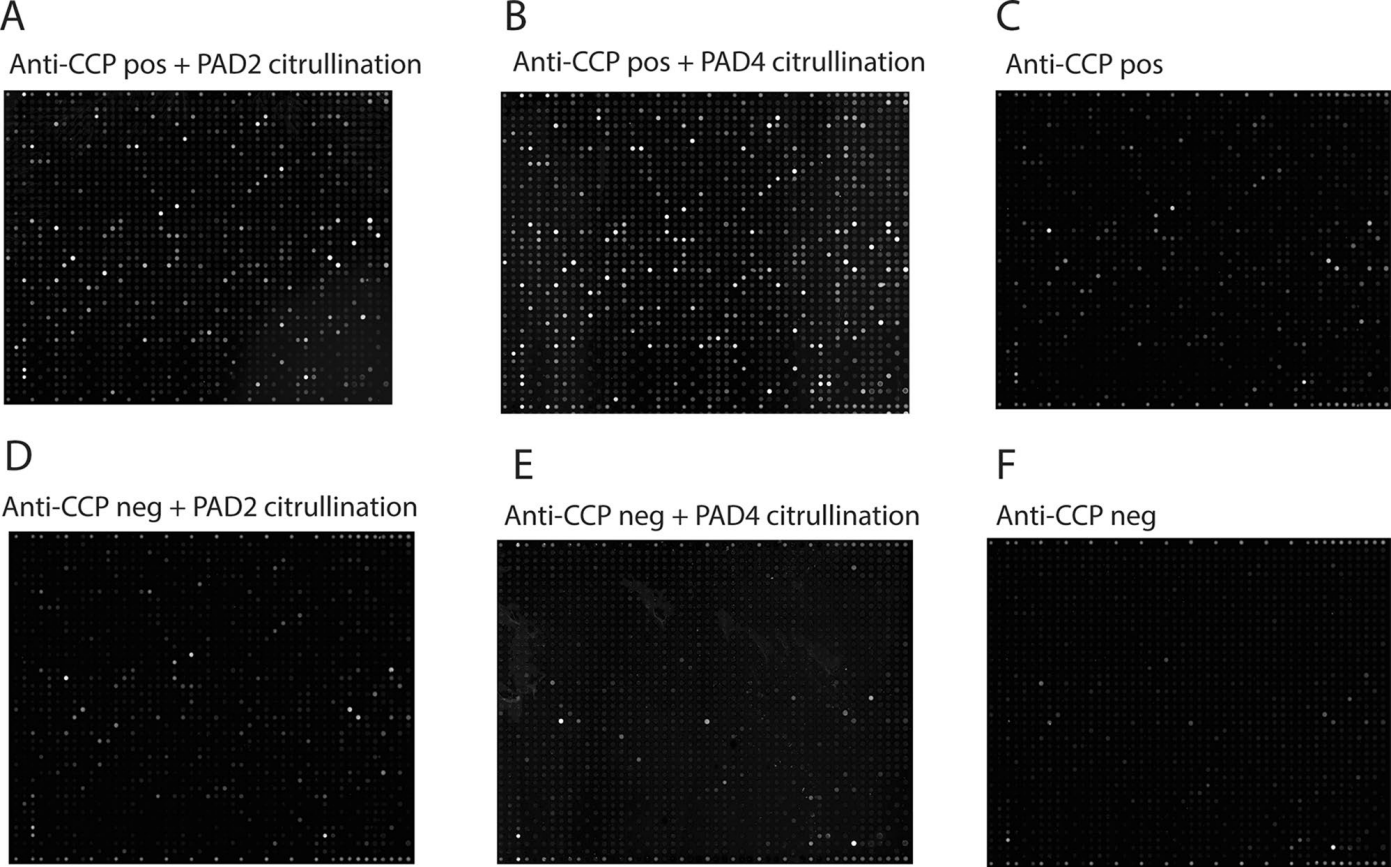
Imaging of autoantibody binding to citrullinated and non-citrullinated protein arrays. Pooled plasma from 15 anti-CCP-positive RA patients or 10 anti-CCP-negative RA patients was diluted 1:200 and added to microarray plates containing 1631 human proteins that had been citrullinated by PAD2 or PAD4 or kept in native form. The binding of IgG antibodies was visualized using Cy3-labelled rabbit anti-human IgG antibodies. (**A**) Slide with proteins citrullinated by PAD2 and incubated with anti-CCP-positive plasma. (**B**) Proteins citrullinated by PAD4 incubated with anti-CCP-positive plasma. (**C**) Native proteins incubated with anti-CCP-positive plasma. (**D**) Proteins citrullinated by PAD2 incubated with anti-CCP-negative plasma. (**E**) Proteins citrullinated by PAD4 incubated with anti-CCP negative plasma (**F**)**.** Native proteins incubated with anti-CCP-negative plasma. (**C**,**F**) have previously been published^20^.

We examined the binding of IgG antibodies in pools of plasma from anti-CCP-positive and anti-CCP-negative RA patients to microarrays containing 1631 human proteins in native form or citrullinated on-slide using PAD2 or PAD4 as catalysts. Figure 1 shows the staining intensity of individual arrays. Data from Fig. 1C,F have previously been published^20^. The PAD enzyme efficiency was tested on fibrinogen and is shown in Supplementary Dataset 1.

### Broad reactivity by low-intensity autoantibodies

**Figure 2:**
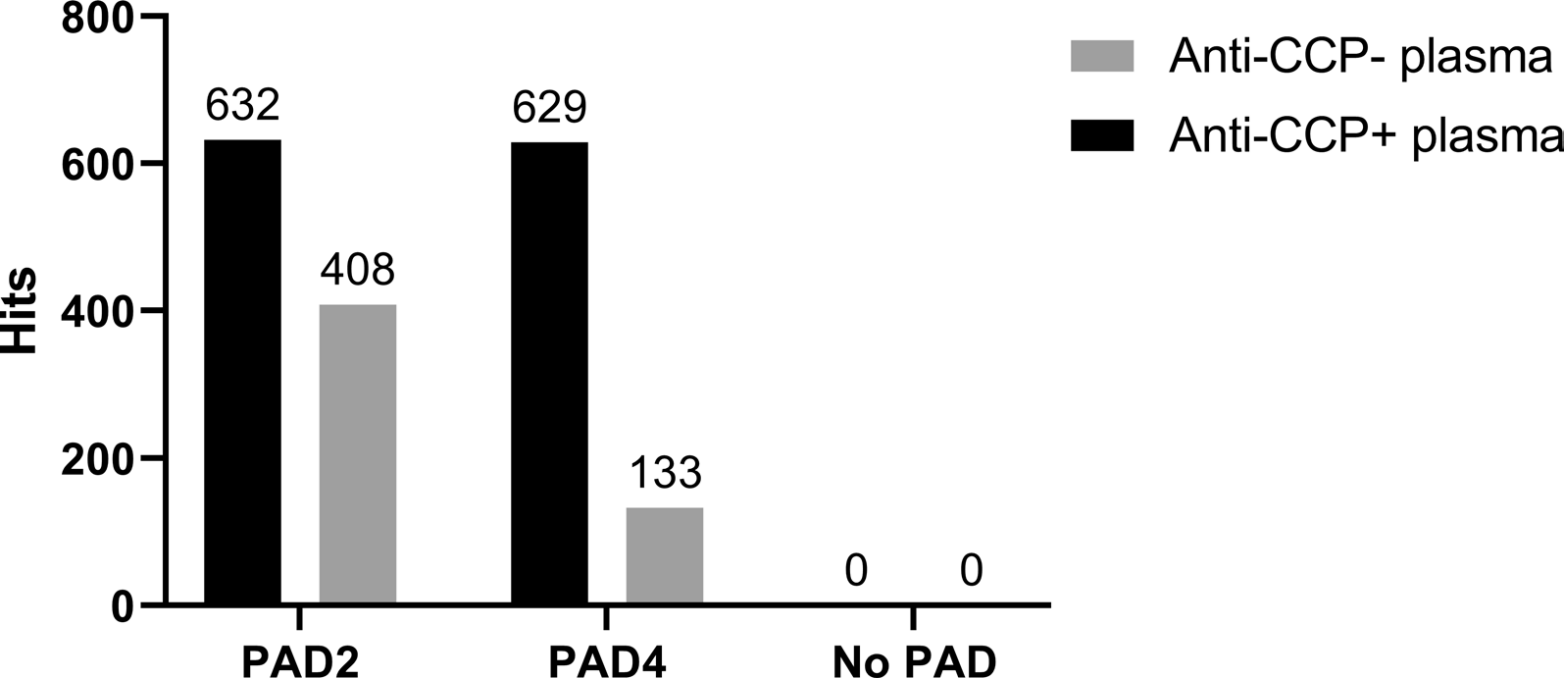
Quantitative analysis of arrayed proteins recognized by autoantibodies. Bar chart showing the number of proteins recognized by autoantibodies to a higher degree than native proteins. Plasma from anti-CCP-positive or anti-CCP-negative RA patients was incubated with Immunome protein microarray slides containing native proteins or proteins citrullinated by PAD2 or PAD4. The number above the bars indicates the number of proteins recognized by autoantibodies under the given conditions (defined as more than two-fold differences in fluorescence intensity compared to native proteins, an intraprotein CV < 15, and a P value < 0.05). Specific targets for autoantibodies are shown in Supplementary Dataset 2.

We observed low reactivity of IgG antibodies against a large number of citrullinated proteins but not against the corresponding native proteins. After incubation with a pool of anti-CCP-positive plasma, 632 proteins showed more than twofold higher binding of IgG after citrullination with PAD2 than in their native form, and the corresponding number was 629 proteins after citrullination with PAD4, suggesting that these proteins were recognized by ACPAs (Fig. 2, Supplementary Dataset 2).

Surprisingly, citrullination also enhanced the binding of IgG autoantibodies to a significant number of proteins when the array was incubated with the anti-CCP-negative plasma pool. This was true for 408 proteins after citrullination with PAD2 and 133 proteins after citrullination with PAD4 (Fig. 2, Supplementary Dataset 2).

PAD2 and PAD4 showed strikingly similar efficiency in generating epitopes recognized by ACPAs. Thus, only one protein, minichromosome maintenance complex-binding protein (C10orf119), was targeted to a higher degree (PAD2/PAD4 ratio: 2.7) by antibodies from anti-CCP-positive plasma when citrullinated by PAD2 compared to PAD4, while only four proteins were targeted to a higher degree when citrullinated by PAD4 (PAD4/PAD2 ratio: approximately 2): PC4 and SFRS1-interacting protein (PSIP1), nucleosome assembly protein 1-like 1 (NAP1L1), protein S100-P (S100P), and nucleosome assembly protein 1-like 4 (NAP1L4).

We also found but few differences between IgG antibody binding to PAD2- and PAD4-citrullinated proteins when the protein array was incubated with the anti-CCP negative plasma pool. Three proteins were targeted to a higher degree when citrullinated by PAD2 than by PAD4 (PAD2/PAD4 ratio: approximately 3): caspase recruitment domain-containing protein 9 (CARD9), 3-phosphoinositide-dependent protein kinase 1 (PDPK1), and protein kinase C and casein kinase substrate in neurons protein 3 (PACSIN3). Only one protein was targeted to a lower degree when citrullinated by PAD2: protein CBFA2T3 (ratio PAD2-citrullinated/PAD4-citrullinated: approximately 0.3).

We did not identify native proteins that were targeted by autoantibodies to a higher degree in the native form than in the citrullinated form. We found a total of 844 citrullinated proteins recognized by ACPAs from RA patients. A list of all identified antigens can be found in Supplementary Dataset 2.

### Binding pattern of autoantibodies from anti-CCP-positive patients

Many of the proteins identified as targets for ACPAs above showed low-intensity staining for IgG antibodies. Proteins that are autoantigens in vivo can be expected to show staining with high intensity; however, to identify proteins that may be autoantigens in vivo, we limited the analysis to include only proteins with z scores > 2 (Tables 1, 2).

**Table 1:** Autoantigens targeted differentially by ACPAs after different citrullination procedures.

Protein	Intra- or extracellular	Anti-CCP positive pool: Ratio: PAD2-citrullinated/native proteins Fold difference	Anti-CCP positive pool: Ratio: PAD4-citrullinated/native proteins Fold difference	Anti-CCP positive pool: Ratio: PAD2-citrullinated /PAD4-citrullinated proteins Fold difference	Anti-CCP negative pool: Ratio: PAD2-citrullinated/native proteins Fold difference	Anti-CCP negative pool: Ratio: PAD4-citrullinated/native proteins Fold difference	Anti-CCP negative pool: Ratio: PAD2-citrullinated/PAD4-citrullinated proteins Fold difference
ACAT2	Intracellular	2.2	3	–	–	–	–
AFF4	Intracellular	5.7	–	–	–	–	–
APPL1	Intracellular	–	–	–	7.2	–	–
BAG3	Intracellular	–	–	–	2.7	2.6	–
C1orf174	Intracellular	-	6.6	–	–	–	–
C21orf2	Intracellular, membrane	2.4	3	–	–	–	–
C21orf33	Intracellular	2.9	4.5	–	2.1	2.1	–
CAMK2G	Intracellular, membrane	2.4	3.4	–	–	–	–
CAMKK2	Intracellular	3.2	–	–	–	–	–
CARD9	Intracellular	-	–	–	4.4	-	3
CARHSP1	Intracellular	2.2	3.1	–	–	–	–
CASS4	Intracellular	15.4		–	–	–	–
CBFA2T3	Intracellular	3.3	4.3	–	–	2.9	0.3
CCM2	Intracellular	3.3	4.3	–	–	–	–
CD96	Membrane	–	–	–	2.8	2.4	–
CEACAM1		–	–	–	–	2.2	–
CNN1	Intracellular	3.3	4.6	–	–	–	–
COMMD3	Both	3	3.8	–	–	–	–
CRISP2	Extracellular, secreted	2	2.8	–	–	–	–
CRYAB		2.2	2.1	–	–	–	–
CT47A1	Intracellular	2	3	–	4.1	2.2	–
CXorf48	Secreted	3.1	3.4	–	–	–	–
DCBLD2	Membrane	–	–	–	2.5	2.1	–
DMRTB1	Intracellular	2.5	3.5	–	–	–	–
DUSP6	Intracellular	3.1	4	–	–	–	–
E6	Intracellular	2.8	3.9	–	–	–	–
EEF1D	Intracellular	2.6	3.5	–	2	2.1	–
EEF1G	Both	2.5	3.2	–	–	–	–
ENO2	Both	–	3.6	–	–	–	–
ESRRG	Intracellular	2.3	3.3	–	–	–	–
ETS2	Intracellular	4.1	–	–	–	–	–
FGFR1_ext	Intracellular, secreted	–	3.5	–	–	–	–
FOXI1	Intracellular	2.1	3.1	–	–	–	–
FOXR2	Intracellular	2.7	2.9	–	–	–	–
FTHL17		2.1	2.8	–	–	–	–
GFAP	Intracellular	2	2.7	–	2.1	–	–
GGPS1		2.3	2.8	–	–	–	–
GNAO1	Intracellular, membrane	2.6	3.5	–	–	–	–
GSTT1	Intracellular	2.1	2.6	–	–	4.6	–
HAGHL	Intracellular	2.5	3.1	–	–	–	–
HDAC1	Intracellular	2.3	3.2	–	–	–	–
HDAC3	Intracellular	2.2	2.9	–	–	–	–
HRAS	Intracellular, membrane	3.5	4.3	–	–	–	–
HSF1	Intracellular	–	3.7	–	–	–	–
ID1	Intracellular, secreted	3.1	4.1	–	–	–	–
IGHG1	Both	2.9	3.8	–	3.1	2.8	–
IKZF1	Intracellular	–	–	–	6.4	–	–
IL1A	Both	2.4	3.4	–	–	–	–
IMPDH1	Intracellular	3	–	–	–	–	–
IRF5	Intracellular	29.5	34.7	–	–	–	–
ITPK1	Intracellular	2.4	3.4	–	–	–	–
KCNIP3	Intracellular, membrane	3.4	4.7	–	–	–	–
KRT8	Intracellular	2.9	3.5	–	–	–	–
LCK	Intracellular, membrane	–	–	–	4.2	3.1	–
LDHB		2.4	2.4	–	–	–	–
LEPREL4	Intracellular	–	2.7	–	–	–	–
LIMS1	Membrane	–	–	–	2.8	3.3	–
MAGEA10	Intracellular	2.6	3.4	–	–	–	–
MAGEB1	Intracellular	5.7	10.1	–	3.5	3.3	–
MAPK8_tv1	Intracellular	2.7	3.9	–	–	–	–
MAPK9	Intracellular	2.9	3.7	–	–	–	–
MAPKAPK3	Intracellular	–	–	–	4.5	–	–
MEF2C	Intracellular	4.9	5.6	–	–	–	–
MIF		2.3	2.4	–	–	–	–
MKNK1	Intracellular, membrane	2.8	2.8	–	–	–	–
MLANA	Intracellular, membrane	2.9	4.3	–	–	–	–
MNAT1	Intracellular	–	–	–	3.2	3.1	–
MOBKL2A	Intracellular	2.6	3.3	–	–	–	–
MPZL2	Membrane	2.4	3.1	–	–	–	–
MX1	Intracellular	2.4	3.2	–	–	–	–
MYCBP		2.1	2.7	–	–	–	–
NAP1L3	Intracellular	–	3.2	–	2.6	2.8	–
NEUROD1		–	2.7	–	–	–	–
NME4	Intracellular	2.4	3.4	–	–	–	–
NPM1	Intracellular	–	5.7	–	–	–	–
NR2E3	Intracellular	2.5	2.8	–	–	–	–
NRBF2		2.2	2.8	–	–	–	–
PACSIN3	Intracellular, membrane	–	–	–	4.5	–	2.5
PCBD	Intracellular	2.3	2.9	–	–	–	–
PDCL3	Intracellular	–	–	–	3.3	2.2	–
PDPK1	Intracellular, membrane	–	–	–	5.5	–	2.8
PKLR	Intracellular, secreted	2.7	3	–	–	–	–
POU2AF1	Intracellular	3.2	4.2	–	–	–	–
PPP1R2P9	Predicted: intracellular	4.4	5.4	–	–	–	–
PRC1	Intracellular	4.1	4.8	–	–	–	–
PRKAR1A	Intracellular, membrane	2.6	2.5	–	–	–	–
PSME2	Both	–	3.8	–	–	–	–
PTPN20A	Intracellular	2.5	3.8	–	10.9	5.5	–
PYCR1		2.6	2.4	–	–	–	–
RBKS	Intracellular	2.5	2.8	–	–	–	–
RBM46	Intracellular	2.2	2.6	–	–	–	–
RBPJ		2	–	–	–	2	–
RNF7	Intracellular	–	3.8	–	–	–	–
RPLP1	Intracellular	2.5	3.4	–	–	–	–
RPS6KA1	Intracellular	–	–	–	3.6	–	–
RPS6KB1	Intracellular	–	–	–	12.2	13.4	–
RQCD1	Intracellular	–	3.9	–	–	–	–
RUFY1	Intracellular	2.7	3.3	–	–	–	–
SDCCAG8	Intracellular	2.6	2.5	–	2.6	2.5	–
SEPT9a	Intracellular	–	9.4	–	–	–	–
SGSM3		2.1	2.5	–	–	–	–
SH3GL1	Intracellular	6.5	5.8	–	3	2.3	–
SKAP1	Intracellular, membrane	3.3	3.9	–	4.5	2.8	–
SPANXN1	Predicted: intracellular	–	3.8	–	–	–	–
SPATA25	Intracellular	2.2	2.7	–	–	–	–
SSBP4	Intracellular	3.1	2.9	–	–	–	–
SSNA1	Intracellular	–	2.2	–	–	–	–
SSX2	Intracellular	3.1	3.8	–	–	–	–
STAT1	Intracellular	3.3	4	–	–	–	–
STAU1	Intracellular	–	20	–	–	–	–
STK3	Intracellular	3.7	4.3	–	–	–	–
TACC1	Intracellular	4.7	4.9	–	–	–	–
TBC1D2	Intracellular	2.7	3.4	–	–	–	–
TEX101	Intracellular, membrane, and secreted	2.6	3.5	–	–	–	–
TFG	Intracellular	3.1	4	–	–	–	–
TKT	Intracellular, secreted	–	3.3	–	–	–	–
TROVE2	Intracellular	–	–	–	–	3.6	–
TSC22D1	Intracellular	2.2	2.6	–	–	–	–
UCKL1	Intracellular	4.4	–	–	–	–	–
USP10	Intracellular	–	–	–	7.7	4	–
VDR	Intracellular	2.9	2.8	–	–	–	–
VIM	Intracellular	–	2.6	–	–	–	–
ZHX2	Intracellular	–	4.1	–	–	–	–
ZNF207	Intracellular	–	–	–	4.4	3.5	–
ZNF496	Intracellular	–	–	–	2.7	2.3	–
ZNHIT3	Intracellular	–	3.2	–	–	–	–

Requirements for inclusion in the table were z score > 2, CV < 15, CI-P < 0.05, fold difference > 2, and a BH corrected P value < 0.05.

*ACAT2* acetyl-CoA acetyltransferase, *AFF4* AF4/FMR2 family member 4, *APPL1* DCC-interacting protein 13-alpha, *BAG3* BAG family molecular chaperone regulator 3, *C1orf174* UPF0688 protein C1orf174, *C21orf2* protein C21orf2, *C21orf33* ES1 protein homolog, *CAMK2B* calcium/calmodulin-dependent protein kinase type II subunit beta, *CAMK2G* calcium/calmodulin-dependent protein kinase type II subunit gamma, *CAMKK2* calcium/calmodulin-dependent protein kinase type II subunit beta, *CARD9* caspase recruitment domain-containing protein 9, *CARHSP1* calcium-regulated heat-stable protein 1, *CASS4* Cas scaffolding protein family member 4, *CBFA2T3* protein CBFA2T3, *CCM2* cerebral cavernous malformations 2 protein, *CD96* T-cell surface protein tactile, *CEACAM1* carcinoembryonic antigen-related cell adhesion molecule 1, *CNN1* Calponin-1, *COMMD3* COMM domain-containing protein 3, *CRADD* death-domain containing protein CRADD, *CRISP2* cysteine-rich secretory protein 2, *CRYAB* alpha-crystallin B chain, *CT47A1* cancer/testis antigen 47A, *CXorf48* cancer/testis antigen 55, *DCBLD2* discoidin, CUB and LCCL domain containing protein 2, *DMRTB1* doublesex- and mab-3-related transcription factor B1, *DUSP6* dual specificity protein phosphatase 6, *E6* protein E6, *EEF1D* elongation factor 1-delta, *EEF1G* elongation factor 1-gamma, *ENO2* gamma-enolase, *ESRRG* estrogen-related receptor gamma, *ETS2* protein C-ets-2, *FGFR1*_*ext* fibroblast growth factor receptor 1, *FOXI1* forkhead box protein I1, *FOXR2* forkhead box protein R2, *FTHL17* ferritin heavy polypeptide-like 17, *GFAP* glial fibrillary acidic protein, *GGPS1* geranylgeranyl pyrophosphate synthase, *GNAO1* guanine nucleotide-binding protein G(o) subunit alpha, *GSTT1* glutathione S-transferase theta-1, *HAGHL* hydroxyacylglutathione hydrolase-like protein, *HDAC1* histone deacetylase 1, *HDAC3* histone deacetylase 3, *HRAS* GTPase HRas, *HSF1* heat shock factor protein 1, *ID1* DNA-binding protein inhibitor ID-1, *IFI35* interferon-induced 35 kDa protein, *IGHG1* immunoglobulin heavy constant gamma 1, *IKZF1* DNA-binding protein Ikaros, *IL1A* Interleukin-1 alpha, *IMPDH1* Inosine-5’-monophosphate dehydrogenase 1, *IRF5* interferon regulatory factor 5, *ITPK1* inositol-tetrakisphosphate 1-kinase, *KCNIP3* calsenilin, *KRT8* keratin, type II cytoskeletal 8, *LCK* tyrosine-protein kinase Lck, *LDHB* L-lactate dehydrogenase B chain, *LEPREL4* endoplasmic reticulum protein SC65, *LIMS1* LIM and senescent cell antigen-like-containing domain protein 1, *MAGEA10* melanoma-associated antigen 10, *MAGEB1* melanoma-associated antigen B1, *MAPK8*_*tv1* mitogen-activated protein kinase 8, *MAPK9* mitogen-activated protein kinase 9, *MAPKAPK3* MAP kinase-activated protein kinase 3, *MEF2C* myocyte-specific enhancer factor 2C, *MIF* acrophage migration inhibitory factor, *MKNK1* MAP kinase-interacting serine/threonine-protein kinase 1, *MLANA* melanoma antigen recognized by T-cells 1, *MNAT1* CDK-activating kinase assembly factor MAT1, *MOBKL2A* MOB kinase activator 3A, *MPZL2* myelin protein zero-like protein 2, *MX1* interferon-induced GTP-binding protein Mx1, *MYCBP* c-Myc-binding protein, *NAP1L3* nucleosome assembly protein 1-like 3, *NEUROD1* neurogenic differentiation factor 1, *NME4* nucleoside diphosphate kinase, *NPM1* nucleophosmin, *NR2E3* photoreceptor-specific nuclear receptor, *NRBF2* nuclear receptor-binding factor 2, *PACSIN3* protein kinase C and casein kinase substrate in neurons protein 3, *PCBD* Pterin-4-alpha-carbinolamine dehydratase, *PDCL3* Phosducin-like protein 3, *PDPK1* 3-phosphoinositide-dependent protein kinase 1, *PKLR* pyruvate kinase PKLR, *POU2AF1* POU domain class 2-associating factor 1, *PPP1R2P9* protein phosphatase inhibitor 2 family member C, *PRC1* protein regulator of cytokinesis 1, *PRKAR1A* cAMP-dependent protein kinase type-I alpha regulatory subunit, *PSME2* proteasome activator complex subunit 2, *PTPN20* tyrosine-protein phosphatase non-receptor type 20, *PYCR1* pyrroline-5-carboxylate reductase 1, mitochondrial, *RBKS* ribokinase, *RBM46* probably RNA-binding protein 46, *RBPJ* recombining binding protein suppressor of hairless, *RNF7* RING-box protein 2, *RPLP1* 60S acidic ribosomal protein P1, *RPS6KA1* ribosomal protein S6 kinase alpha-1, *RPS6KB1* ribosomal protein S6 kinase beta-1, *RQCD1* CCR4-NOT transcription complex subunit 9, *RUFY1* RUN and FYVE domain-containing protein 1, *SDCCAG8* serologically defined colon cancer antigen 8, *SEPT9a* Septin-9a, *SGSM3* small G protein signaling modulator 3, *SH3GL1* endophilin-A2, *SKAP1* Src kinase-associated phosphoprotein 1, *SPANXN1* sperm protein, *SPATA25* spermatogenesis-associated protein 25, *SSBP4* single-stranded DNA-binding protein 4, *SSNA1* Sjoegren syndrome nuclear autoantigen 1, *SSX2* protein SSX2, *STAT1* signal transducer and activator of transcription 1-alpha/beta, *STAU1* double-stranded RNA-binding protein Staufen homolog 1, *STK3* serine/threonine-protein kinase 3, *TACC1* transforming acidic coiled-coil-containing protein 1, *TBC1D2* TBC1 domain family member 2A, *TEX101* testis-expressed protein 101, *TFG* protein TFG, *TKT* transketolase, *TROVE 2* 60 kDa SS-A/Ro ribonucleoprotein, *TSC22D1* TSC22 domain family protein 1, *UCKL1* uridine-cytidine kinase like 1, *USP10* ubiquitin carboxyl-terminal hydrolase 10, *VDR* vitamin D3 receptor, *VIM* vimentin, *ZHX2* zinc fingers and homeoboxes protein 2, *ZNF207* BUB3-interacting and GLEBS motifc containing protein, *ZNF496* zinc finger protein 496 , *ZNHIT3* zinc finger HIT domain-containing protein 3.

–: did not fulfill the inclusion criteria as stated above.

**Table 2:** Citrullinated autoantigens targeted by autoantibodies.

Proteins	Intra- or extracellular	PAD2-citrullinated proteins Ratio: anti-CCP positive/anti-CCP negative	PAD4-citrullinated proteins: Ratio: anti-CCP positive/anti-CCP negatives
ACAT2	Intracellular	9.2	12.7
AFF4	Intracellular	2.7	–
ALDOA	Intracellular, secreted	9.1	6
C1orf174	Intracellular	–	6.9
C21orf2	Intracellular, membrane	3.9	4.4
C21orf33	Intracellular	2.1	3.3
CAMK2G	Intracellular, membrane	3.7	4.8
CAMKK2	Intracellular	4.3	–
CARHSP1	Intracellular	12.5	13.3
CASS4	Intracellular	4.5	–
CBFA2T3	Intracellular	2.6	–
CCM2	Intracellular	3.1	5.1
CNN1	Intracellular	5.8	7.8
COMMD3	Both	6	9.7
CRISP2	Extracellular, secreted	3	4.2
CRYAB	Intracellular, membrane	6	4.3
CT47A1	Intracellular	0.4	–
CXorf48	Secreted	5.5	5.9
DMRTB1	Intracellular	4.8	6.1
DUSP6	Intracellular	4.9	6.7
E6	Intracellular	7	12.4
EEF1D	Intracellular	3	3.9
EEF1G	Both	6.1	7.2
ENO2	Both	–	8.1
ESRRG	Intracellular	7.3	9
ETS2	Intracellular	3.8	–
FGFR1_ext	Intracellular, secreted	–	3.9
FOXI1	Intracellular	2.3	3.2
FOXR2	Intracellular	5.9	5.8
FTHL17	Intracellular	4.5	5
GFAP	Intracellular	2	3.9
GGPS1	Intracellular	6.4	7.4
GNAO1	Intracellular, membrane	3.3	5
HAGHL	Intracellular	2.5	3.3
HDAC1	Intracellular	3	4.3
HDAC3	Intracellular	3.7	5.7
HRAS	Intracellular, membrane	3.8	5.6
HSF1	Intracellular	–	9.6
ID1	Intracellular, secreted	5.7	6.9
IL1A	Both	3.7	4.6
ILF2	Intracellular	4.3	4
IMPDH1	Intracellular	5.4	–
IRF5	Intracellular	5.1	6.9
ITPK1	Intracellular	3.4	4.6
KCNIP3	Intracellular, membrane	7.2	10.3
KRT15	Both, exosome	3.2	2.3
KRT19	Both, exosome	3.3	–
KRT8	Intracellular	9.9	12.4
LDHB	Intracellular	6.8	6.8
LEPREL4	Intracellular	3.3	5
MAGEA10	Intracellular	6.1	7.5
MAGEB1	Intracellular	2.8	5.2
MAPK8_tv1	Intracellular	5.6	11.2
MAPK9	Intracellular	3.9	6.6
MEF2C	Intracellular	4	6.9
MIF	Both	10.9	7.7
MKNK1	Intracellular, membrane	5.5	5.6
MLANA	Intracellular, membrane	6	8
MOBKL2A	Intracellular	7.4	11.7
MPZL2	Membrane	4.4	5
MX1	Intracellular	4.4	6.8
MYCBP	Intracellular	9.2	9.6
NEUROD1	Intracellular	–	5.8
NME4	Intracellular	6.4	6.8
NPM1	Intracellular	–	5.6
NR2E3	Intracellular	5.7	6.5
NRBF2	Intracellular	7	7.6
NUBP2	Intracellular	5.2	3.5
ODC1	Intracellular	6.5	5.8
PCBD	Intracellular	7.3	9.1
PKLR	Intracellular, secreted	4.1	4.8
POU2AF1	Intracellular	4.6	6.9
PPP1R2P9	Predicted: intracellular	4.5	5.9
PRC1	Intracellular	8.6	8.1
PRKAR1A	Intracellular, membrane	7.8	6.9
PSME2	Intracellular	–	8.2
PYCR1	Intracellular	7.5	5.9
RBKS	Intracellular	7.4	9.1
RBM46	Intracellular	8.1	8.1
RNF7	Intracellular	–	5
RPLP1	Intracellular	4.4	5
RQCD1	Intracellular	–	7.1
RUFY1	Intracellular	4.8	5.5
SEPT9a	Intracellular	–	6.6
SGSM3	Intracellular, membrane	4.1	4.5
SH3GL1	Intracellular	3.7	4.4
SPANXN1	Predicted: intracellular	–	6.5
SPANXN2	Intracellular	3.1	2.6
SPATA25	Intracellular	4.7	4.8
SSBP4	Intracellular	8	7.9
SSNA1	Intracellular	–	11.1
SSX2	Intracellular	5.8	7.1
STAT1	Intracellular	5.6	6.3
STAU1	Intracellular	–	6.3
STK3	Intracellular	4.9	7.3
TACC1	Intracellular	3.8	3.8
TBC1D2	Intracellular	4.1	5
TEX101	Intracellular, membrane, and secreted	4.7	–
TFG	Intracellular	5.1	7.1
TKT	Intracellular, secreted	–	5.8
TPM1	Intracellular, membrane	–	4.1
TSC22D1	Intracellular	5	5.6
TSPY3	Intracellular	6.4	4
UCKL1	Intracellular	3.9	–
VDR	Intracellular	6.9	7.6
VIM	Intracellular	4.2	4.8
ZHX2	Intracellular	–	5.2
ZNHIT3	Intracellular	–	5.3

Results are listed as fold differences calculated from the relative fluorescence unit value and are statistically significant.

Requirements for inclusion in the table were Z-score > 2, CV < 15, CI-P < 0.05, fold difference > 2, and a BH corrected P value < 0.05.

*ALDOA* fructose-biphosphate aldolase A, *ILF2* interleukin enhancer-binding factor 2, *KRT15* keratin, type I cytoskeletal 15, *KRT19* keratin, type I cytoskeletal 19, *NUBP2* cytosolic Fe-S cluster assembly factor NUBP2, *ODC1* ornithine decarboxylase, *PSME3* proteasome activator complex subunit 3, *SPANXN2* sperm protein associated with the nucleus on the X chromosome N2, *SSB* lupus La protein, *TPM1* tropomyosin alpha-1 chain, *TSPY3* testis-specific Y-encoded protein 3, *VEGFB* vascular endothelial growth factor B. The remaining names are listed in the footer of Table 1.

–: did not fulfill the inclusion criteria as stated above.

After incubation of the arrayed proteins with the anti-CCP-positive plasma pool, two well-established autoantigens in RA fulfilled this criterion: vimentin and keratin 8. For both proteins, the binding of IgG autoantibodies increased markedly when they were citrullinated by PAD4 compared to native proteins, while the same only applied to keratin 8 after citrullination with PAD2 (Table 1). Irrespective of whether PAD2 or PAD4 was used for citrullination, more antibody binding was observed after incubation with the anti-CCP-positive plasma pool than after incubation with the anti-CCP-negative plasma pool (Table 2).

The proteins that showed the greatest increase in autoantibody capture after citrullination with PAD2 compared to native proteins were interferon-induced 35 kDa protein (IRF5; 29.5-fold increase after citrullination), cas scaffolding protein family member 4 (CASS4; 15.4-fold increase), and endophilin-A2 (SH3GL1; 6.5-fold increase). The proteins with the greatest change in autoantibody capture after citrullination with PAD4 were IRF5 (34.7-fold increase), double-stranded RNA-binding protein Staufen homolog 1 (STAU1; 20.0-fold increase), and melanoma-associated antigen B1 (MAGEB1; 10.1-fold increase).

### Binding pattern of autoantibodies from anti-CCP-negative patients

We next examined the binding of autoantibodies contained in the plasma pool from anti-CCP-negative patients to citrullinated and native proteins. Even after exclusion of proteins with z scores < 2, we identified several proteins that showed increased IgG autoantibody binding after citrullination. This applied to 29 proteins after citrullination by PAD2 and 26 proteins after citrullination by PAD4 (Table 1).

### Comparison between anti-CCP-positive and anti-CCP-negative plasma

Finally, we examined the binding of IgG autoantibodies to the protein array after incubation with the anti-CCP-positive versus the anti-CCP-negative plasma pool (Table 2). When PAD2 was used for citrullination, 91 proteins showed more than twofold higher binding of autoantibodies when incubated with anti-CCP-positive plasma than with anti-CCP-negative plasma. After citrullination with PAD4, the corresponding number was 98 proteins. The most significant differences were observed for calcium-regulated heat-stable protein 1 (CARHSP1, ratio anti-CCP-positive plasma/anti-CCP-negative: 12.5), macrophage migration inhibitory factor (MIF; ratio 10.9), and keratin type II cytoskeletal 8 (KRT8; ratio 9.9) when PAD2 was used for citrullination. When PAD4 was used for citrullination, the greatest anti-CCP-positive/anti-CCP-negative ratios were observed for calcium-regulated heat-stable protein 1 (CARHSP1, ratio 13.3), acetyl-CoA acetyltransferase (ACAT2, ratio: 12.7), and protein E6 (E6, ratio 12.4).

## Discussion

We performed a high-throughput high-density protein microarray analysis on pools of plasma from 15 anti-CCP-positive and 10 anti-CCP-negative RA patients to identify proteins recognized by IgG autoantibodies before and after citrullination. The method proved successful, and we provide here a list of 844 out of 1631 arrayed proteins that were recognized by autoantibodies after citrullination, i.e. recognized by ACPAs. To our knowledge, this is the largest number of proteins identified as potential targets of ACPAs to date. Previous studies have used other types of citrullinated protein arrays to investigate ACPA reactivity but have focused on a single or fewer citrullinated proteins, usually on known RA antigens such as vimentin, fibrinogen, and alpha-enolase or used processed sample material^11,22–29^. This is the first investigation of autoantibody reactivity against citrullinated proteins on the KREX protein array platform using pure plasma samples from RA patients that we are aware of. Although we demonstrate here that more than 800 proteins can be recognized by ACPAs, they are not necessarily autoantigens in vivo, where several requirements must be met for citrullination to occur: the protein must localize to the same compartment as PAD2 or PAD4 and requirements to pH level, reducing conditions and calcium concentration should be met^30–33^. More research is needed to clarify which of the proteins shown to bind ACPAs under the optimal conditions used here in vitro also do so in vivo and elaboratory validation experiments are critical in this regard.

Per se, the high numbers of identified ACPA targets suggest that PAD enzymes are promiscuous in generating citrullinated neoepitopes recognized by ACPAs. On the other hand, approximately half of the proteins in the protein array used here were not recognized by ACPA, suggesting either that those proteins lack surface-exposed arginine residues or that they lack citrullination motifs for PADs.

Many of the abovementioned proteins that bound IgG autoantibodies showed low staining intensity. ACPAs appear to consist of a pool of either specific or cross-reactive antibodies, and it can be speculated that the low staining intensity is a result of cross-reactive antibodies^34^. The literature on monoclonal ACPAs shows extensive cross-reactivity, especially if glycine is present in the + 1 position of citrulline^35,36^. This fact may be important to consider in any multiplex ACPA assay using on-array citrullination so that there is complete control of which epitopes are citrullinated and which are not. Proteins that are potential autoantigens in vivo are likely to have relatively high affinity and/or concentrations, so in an effort to narrow down the list to potential genuine autoantigens, we implemented an additional filtration using z score (cut-off > 2) that excluded low-intensity antigens. Approximately 100 proteins in anti-CCP-positive plasma were identified. Among them was vimentin, a well-known autoantigen in RA. Other proteins showed strong increases in IgG binding intensity after citrullination, e.g., IRF5, CASS4, SH3GL1, and STAU1. Further studies are needed to determine whether the citrullinated forms of these proteins contain T-cell epitopes in addition to being targeted by ACPAs.

Interestingly, we also identified a rather large number of proteins recognized by autoantibodies from anti-CCP-negative individuals. This has been shown several times before and may demonstrate a subgroup of RA patients not identified using traditional serological testing^37–39^. Furthermore, this supports the conclusion of Wagner and colleagues that the commonly used commercial anti-CCP assays fail to identify some ACPA-positive RA patients (at least 10% in the authors setup)^40^. ACPA-positive and ACPA-negative RA have quite different pathogenesis^6,7^, and when future treatment targeting ACPA-positive RA specifically (e.g., PAD inhibitors) becomes available, protein arrays such as the one employed here may discriminate ACPA-positive and ACPA-negative RA better than the anti-CCP test. Another explanation why we identify several proteins recognized by autoantibodies from anti-CCP negative patients may be due to the implication of citrullination and not the citrullinated epitope. As already mentioned, the citrullination process results in conformational changes of the proteins which may lead to recognition of unmodified proteins from the anti-CCP negative patient pool and not necessarily recognition of the citrullinated epitope.

The relative efficiency of PAD2 and PAD4 in generating epitopes recognized by ACPAs has been a matter of some controversy. One study showed that at high antibody titers (1:250 and 1:1000) but not low titers (1:40 and 1:100), ACPAs preferentially bind to fibrinogen citrullinated by PAD4, while we have previously reported that PAD2 and PAD4 are equally efficient in generating epitopes for the binding of ACPAs to fibrinogen and alpha-enolase^10,11^. In a similar setup, we previously showed that PAD4 was the dominant isoform in generating ACPA-binding sites in histone H3^11^. At the serum dilution used in the present study (1:200), the staining IgG ACPAs was equally intense when proteins were citrullinated by PAD2 and PAD4, except for only four proteins out of 1631 proteins.

The protein array methodology to investigate posttranslationally modified epitopes may not only be relevant for RA but may also be used in diseases where autoantibodies against other modified proteins have been shown, such as oxidized proteins in type 1 diabetes or autoantigens phosphorylated during stress-induced apoptosis in systemic lupus erythematosus^5,41,42^. Furthermore, it may be relevant to investigate at risk individuals to compare citrullination profiles or investigate other PAD enzymes such as the *P. gingivalis* PAD enzyme, which has been proposed to trigger RA even though conflicting studies exist^43,44^.

A limitation to the current study is that the Immunome protein arrays were not specifically enriched for antigens of particular relevance for RA, i.e. proteins that are present in joints or proteins that have previously been identified as autoantigens in RA, although numerous prominent ribonuclear proteins and other well-known autoantigens are present on the arrays. The development of focused arrays containing such proteins would be a natural next step. Another limitation is the use of plasma pools rather than individual plasma samples. Using individual samples, we could compare specific clinical phenotypes to autoantibody patterns or demonstrate the potential of subdifferentiation of patients based on their autoantibody profile^45–48^. This first study of its kind was merely a proof-of-concept study; it proves that further development of the technique is warranted.

## Conclusion

We present a list of 844 citrullinated proteins recognized by ACPAs from RA patients. We demonstrate that PAD2 and PAD4 are equally efficient at generating binding sites for ACPAs. We present a list of approximately 100 potential autoantigens in RA, and we suggest that the pattern of autoantibody recognition may form a basis for subgrouping of anti-CCP-positive RA patients and anti-CCP-negative patients that rightfully should be considered ACPA-positive. The next steps in the development of the technique should be the production of arrays with RA-associated antigens and comparison of ACPA reactivity patterns with clinical phenotypes.

## Materials and methods

### Collection of patient plasma

Plasma samples were obtained from 10 anti-CCP-negative RA patients and 15 anti-CCP-positive RA patients. Patient data can be seen in Table 3. Plasma from anti-CCP-negative and anti-CCP-positive RA patients was pooled separately before protein array analysis. Individual patient response data have previously been published, and we have previously used the same patient cohorts in another study to investigate autoantibody reactivity against native autoantigens^11,20^. RA patients fulfilled the American College of Rheumatology and European League Against Rheumatism criteria for the diagnosis of RA^49^. Plasma was isolated from peripheral venous blood and drawn into BD Vacutainers containing EDTA (BD, Plymouth, UK). The use of patient samples was approved by the local ethics committee of the Institute of Rheumatology in Prague, Czech Republic, and written informed consent was obtained from all patients before initiation of the study (June 26, 2012, No. 3294/2012). All methods were performed according to relevant guidelines and regulations and in accordance with the Declaration of Helsinki.

**Table 3:** Patient data from included participants.

	Anti-CCP-positive (n = 15)	Anti-CCP-negative (n = 10)
Age (years)	52.8 ± 15.6 (27–84)	53.8 ± 17.1 (34–81)
Gender	11 females and 4 males	5 females and 5 males
Anti-CCP (median)	891 mU [interquartile range 784]	3.6 mU [interquartile range 10]
DAS28-ESR	5.0 ± 1.4	3.7 ± 0.8
DAS28-CRP	4.7 ± 1.2	4 ± 1.2
CRP (mg/L)	29.4 ± 31.8	12.6 ± 8.9
RF IgM	91.7 ± 126.3	21.6 ± 19.7
Treatment	14 receiving csDMARDs and 4 also receiving bDMARDs	7 receiving csDMARDs and 1 receiving only bDMARDs

### Sample preparation for protein array analysis

**Figure 3:**
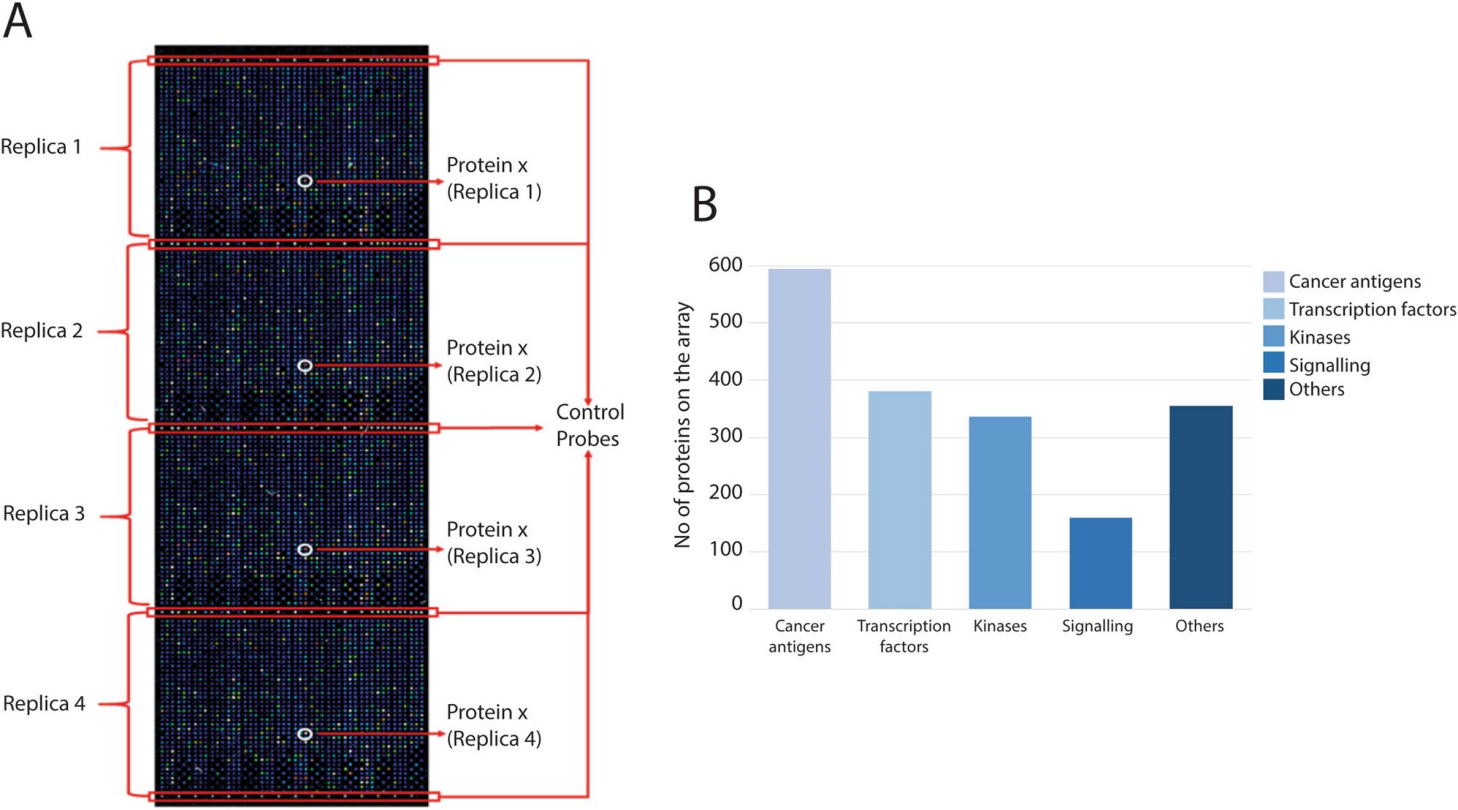
Immunome slide design and protein categories on the protein microarray. (**A**) The Immunome protein microarray consists of four identical subarrays, each containing 1631 different proteins in addition to 11 control proteins (BCCP, BSA, Cy3BSA, IgA, IgG, IgM, and four different control probes for the BCCP tag acting as negative controls). The control proteins are located between the subarrays. (**B**) The proteins spotted on the microarray represent different protein groups, such as cancer-associated antigens, transcription factors, kinases, and signaling proteins. The number of proteins in each category is shown. *BCCP* biotin carboxyl carrier protein, *BSA* bovine serum albumin.

The Immunome (v1) protein microarray (Sengenics, Singapore) consists of 1631 proteins, in addition to several control proteins, spotted in quadruplicates, allowing assessment of spot-to-spot variation and across-slide variation in background intensity (Fig. 3A). The microarray consists of a variety of different proteins representing different categories, such as cancer-associated antigens, transcription factors, kinases, and other proteins involved in inflammation and cell signaling (Fig. 3B).

Each protein in the array is coupled to a biotin carboxyl carrier protein tag, which ensures correct three-dimensional folding during expression. Four slides were carefully transferred to different quadriperm chambers (Greiner BioOne, Kremsmünster, Austria) containing different citrulline reaction buffers consisting of 1.2 µg/mL recombinant human PAD2 or PAD4 (Cayman Chemicals, Ann Arbor, MI, USA); 1 mM 1,4-dithiothreitol (DTT), 10 mM CaCl, and 100 mM Tris–HCl and incubated at 37 °C for 3 h while shaking under horizontal rotation at 50 rpm (IKA, Germany, Königswinter). The slides were washed two times using cold serum albumin buffer (SAB) containing 0.1% Triton X-100 and 0.1% bovine serum albumin (BSA) in phosphate-buffered saline (PBS) and placed in a new quadriperm chamber. Additionally, two slides following the same procedure, but without the addition of PAD enzymes, were used.

Four milliliters of diluted pooled plasma (1:200) from anti-CCP-positive or anti-CCP-negative RA patients was added to the new chambers and incubated at 20 °C for 2 h at 50 rpm. The slides were washed using SAB buffer and added to a new quadriperm chamber. The binding of IgG antibodies was detected using Cy3-conjugated (GE Healthcare, Chicago, Ill, USA) polyclonal rabbit anti-human IgG (Dako, Santa Clara, CA, USA) diluted 1:1000 (v/v) in SAB buffer. The slides were covered in tinfoil and incubated at 20 °C for 2 h at 50 rpm. Finally, the slides were washed twice in SAB buffer and three times in ultrapure water followed by centrifugation at 240*g* for 5 min to dry the slides. Slides were stored at room temperature and scanned within 24 h.

### Protein array imaging

The intensity of the individual spots was measured using a microarray laser scanner (Innoscan 710AL, Innopsys, Carbonne, France) using Mapix software (Ver. 8.2.2, Innopsys). The scan settings were as follows: 532 nm laser with low laser power (5 V), PMT gain at 60%, 5 µm resolution, and a scan speed of 35 px/s. Spotxel (SICASYS ver. 1.7.6) was used to automatically annotate each protein on the slide. Semiautomatic array alignment was used to specify the location of each spot. The median pixel intensity for each spot was used to eliminate the effect of outliers. Background intensity levels were extracted from the intensity of the adjacent spot. The data were exported as CSV files, and further data analysis was performed in R (Ver. 1.1.456, R Core Team).

### Protein array quantitation data analysis

Raw intensities were normalized using a combination of quantile and intensity-based normalization^50^. Based on the normalized intensity levels, a z score, percent coefficient of variation (CV%), and Chebyshev inequality precision (CI-P) were calculated for each protein. An intraprotein CV% cutoff of < 15 was applied to ensure high reproducibility between the same protein spots (n = 4) on each microarray and to demonstrate an equal degree of citrullination. A CI-P cutoff of < 0.05 was applied to ensure that the identified intensities did not belong to the negative control distribution. We applied a z score with a cutoff of > 2 to discard low RFU intensities. Two-sample t tests with Benjamini–Hochberg FDR were performed to identify any statistically significant changes between the positive spots and the corresponding spots on the other slides. Finally, ratios for the statistically significantly changed expressions were calculated, and fold differences below 2 were discarded.

### Mass spectrometry sample preparation

Fibrinogen (Cayman Chemical) was incubated for 3 h at 37 °C in citrulline reaction buffer containing 1.2 µg/mL PAD2 or PAD4 (Cayman Chemicals), 1 mM DTT, 10 mM CaCl, and 100 mM Tris–HCl to citrullinate fibrinogen. Digestion of fibrinogen (Cayman Chemical) was performed using filter-aided sample preparation^51^. Briefly, samples were transferred to Amicon Ultra 0.5 Centrifugal filters 10 kDa (Merck Millipore, MA, USA) containing 0.5% SDC in 50 mM triethylammonium bicarbonate (TEAB) buffer were centrifuged at 14,000*g* for 15 min. Next, the samples were reduced and alkylated by incubating in 10 mM tris(2-carboxyethyl)phosphine hydrochloride and 50 mM chloroacetamide for 30 min at 37 °C. Samples were washed in 0.5% sodium deoxycholate in 50 mM TEAB, and each wash was followed by centrifugation at 14,000*g* for 15 min at 20 °C. Next, samples were digested using 1 µg trypsin/100 µg sample protein in 0.5% SDC in TEAB and incubated overnight at 37 °C. Peptides were eluted by centrifugation at 14,000*g* for 15 min followed by the addition of 200 µl TEAB buffer and another centrifugation step. Next, the peptides were isolated by phase separation using ethyl acetate and acidified by trifluoroacetic acid. Phase separation was repeated two times, and the aqueous phase containing the peptides was recovered. All samples were dried down and stored at − 20 °C until the time of analysis.

### Ultra-performance liquid chromatography tandem mass spectrometry

Fibrinogen samples were rehydrated in 2% acetonitrile and 0.1% formic acid. Protein concentration was measured using a DeNovix spectrophotometer DS-11 FX+ (DeNovix, Wilmington, Del, USA), and 0.4 µg was loaded per sample. Peptides were separated by reverse-phase liquid chromatography on a UPLC system (Dionex RSLX, Thermo Scientific), ionized by a nanoelectrospray ion source (CaptiveSpray, Bruker Daltonics), and analyzed using a timsTOF PRO mass spectrometer (Bruker Daltonics, Bremen, Germany). Samples were injected directly onto a C18 reversed-phase column (IonOpticks, 25 cm × 75 µm ID, 1.6 µm C18) kept at 40 °C. The peptides were eluted with a constant flow rate of 400 nL/min using solvent A (0.1% formic acid in water) and solvent B (acetonitrile with 0.1% formic acid) with a total runtime of 60 min. The gradient was as follows: 0–16 min at 2% B, 16–45 min at 5% B, 45–48 at 35% B, 48–52 min at 95% B, 52–60 min at 2% B. Raw files were loaded into PEAKS (Bioinformatics Solution Inc, v. 10.5) and followed the standard analysis pipeline with the addition of citrulline as a variable modification.

### Ethics approval and consent to participate

The use of patient samples was approved by the local ethics committee of the Institute of Rheumatology in Prague, Czech Republic, and written informed consent was obtained from all patients before initiation of the study (June 26, 2012, No. 3294/2012).

## Supplementary Information

Supplementary Information 1.

Supplementary Information 2.

Supplementary Information 3.

## Data Availability

The microarray raw data are available from the corresponding author on reasonable request. The mass spectrometry proteomics data generated during the current study are available from the ProteomeXchange Consortium via the partner repository with the dataset identifier PXD024955^52^.

## Abbreviations

RA: Rheumatoid arthritis
ACPA: Anti-citrullinated protein antibody
PAD: Protein arginine deiminase
anti-CCP: Anti-cyclic citrullinated peptide
CV%: Percent coefficient of variation
CI-P: Chebyshev inequality precision
BCCP: Biotin carboxyl carrier protein
