# Supplementary figures and images for: Identification of potential autoantigens in anti-CCP-positive and anti-CCP-negative rheumatoid arthritis using citrulline-specific protein arrays
Source: Sci Rep. 2021 Aug 27;11:17300. doi: 10.1038/s41598-021-96675-z (PMC8397748; doi:10.1038/s41598-021-96675-z)

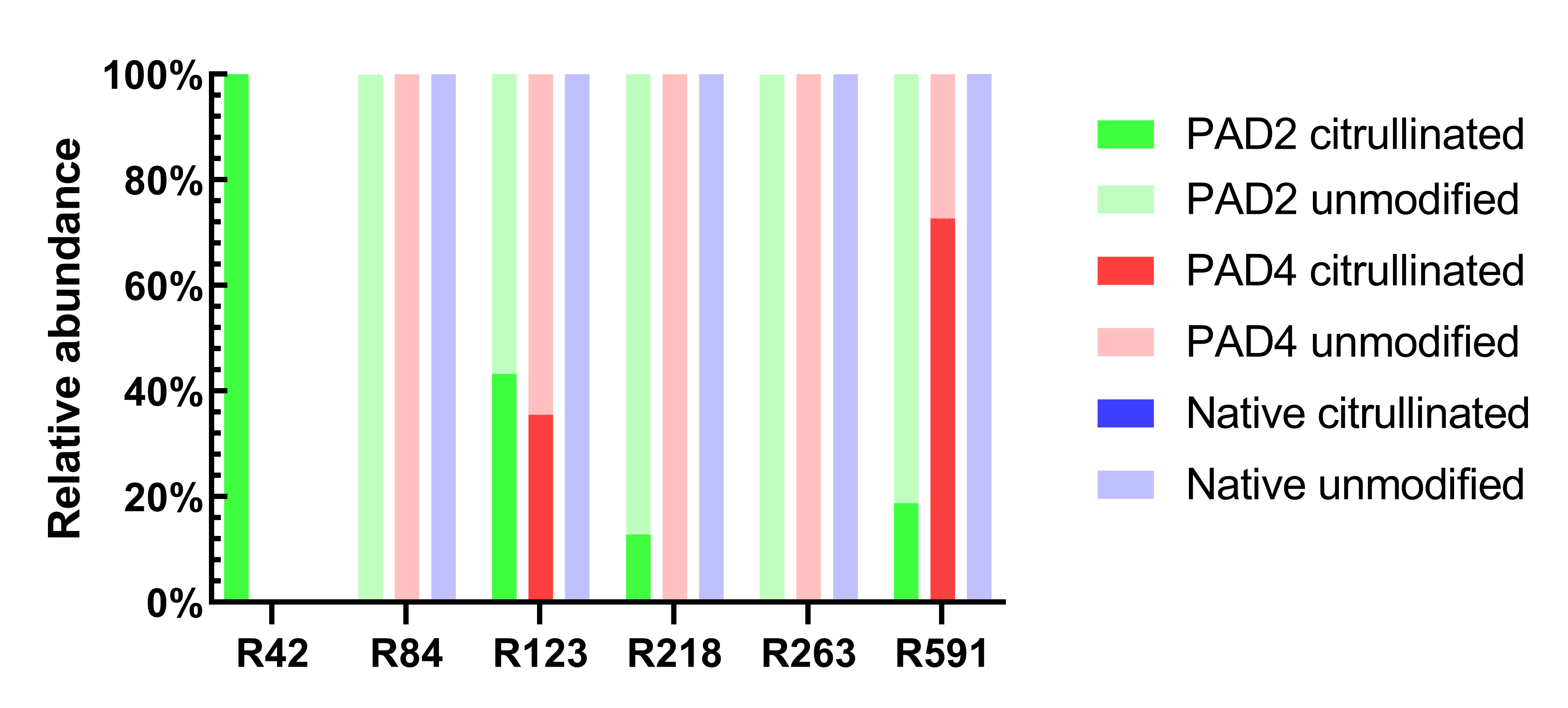

Supplement: Supplementary file 1 — Supplementary Information 1. [file 41598_2021_96675_MOESM1_ESM.tif]
