# Supplementary material for: Identification of potential autoantigens in anti-CCP-positive and anti-CCP-negative rheumatoid arthritis using citrulline-specific protein arrays
Source: Sci Rep. 2021 Aug 27;11:17300. doi: 10.1038/s41598-021-96675-z (PMC8397748; doi:10.1038/s41598-021-96675-z)
Supplement: Supplementary file 3 — Supplementary Information 3. [file 41598_2021_96675_MOESM3_ESM.docx]

**Identification of potential autoantigens in anti-CCP-positive and anti-CCP-negative rheumatoid arthritis using citrulline-specific protein arrays**

Thomas B. G. Poulsen, MSc^1,2^, Dres Damgaard, MSc, Ph. D.^3^, Malene Møller Jørgensen, Ph. D^4,5^, Ladislav Senolt^6^, Jonathan M. Blackburn, Ph. D^7^, Claus H. Nielsen MD, MSc, Ph. D^3#^, Allan Stensballe Ph. D^1*#^

**Supplementary Dataset 1. Relative abundance of citrullinated and unmodified sites on fibrinogen alpha chain.** Bold colors refer to citrullinated sites while transparent colors refer to unmodified sites. Fibrinogen was incubated with either PAD2, PAD4 or no PAD enzyme corresponding to the 3 categories in the figure (PAD2, PAD4, native).
